# Experimental Evolution of a Plant Pathogen into a Legume Symbiont

**DOI:** 10.1371/journal.pbio.1000280

**Published:** 2010-01-12

**Authors:** Marta Marchetti, Delphine Capela, Michelle Glew, Stéphane Cruveiller, Béatrice Chane-Woon-Ming, Carine Gris, Ton Timmers, Véréna Poinsot, Luz B. Gilbert, Philipp Heeb, Claudine Médigue, Jacques Batut, Catherine Masson-Boivin

**Affiliations:** 1 Laboratoire des Interactions Plantes Micro-organismes (LIPM), UMR CNRS-INRA 2594/441, Castanet-Tolosan, France; 2 CNRS-UMR 8030, Evry, France; 3 Laboratoire des IMRCP, UMR UPS/CNRS 5623, Toulouse, France; 4 CNRS, UPS, EDB (Laboratoire évolution et Diversité Biologique), UMR5174, Université de Toulouse, Toulouse, France; Massachusetts Institute of Technology, United States of America

## Abstract

Rhizobia are phylogenetically disparate α- and β-proteobacteria that have achieved the environmentally essential function of fixing atmospheric nitrogen in symbiosis with legumes. Ample evidence indicates that horizontal transfer of symbiotic plasmids/islands has played a crucial role in rhizobia evolution. However, adaptive mechanisms that allow the recipient genomes to express symbiotic traits are unknown. Here, we report on the experimental evolution of a pathogenic *Ralstonia solanacearum* chimera carrying the symbiotic plasmid of the rhizobium *Cupriavidus taiwanensis* into *Mimosa* nodulating and infecting symbionts. Two types of adaptive mutations in the *hrpG*-controlled virulence pathway of *R. solanacearum* were identified that are crucial for the transition from pathogenicity towards mutualism. Inactivation of the *hrcV* structural gene of the type III secretion system allowed nodulation and early infection to take place, whereas inactivation of the master virulence regulator *hrpG* allowed intracellular infection of nodule cells. Our findings predict that natural selection of adaptive changes in the legume environment following horizontal transfer has been a major driving force in rhizobia evolution and diversification and show the potential of experimental evolution to decipher the mechanisms leading to symbiosis.

## Abstract 2

Following acquisition of a rhizobial symbiotic plasmid, adaptive mutations in the virulence pathway allowed pathogenic *Ralstonia solanacearum* to evolve into a legume symbiont under plant selection.

## Author Summary

Most leguminous plants can form a symbiosis with members of a group of soil bacteria known as rhizobia. On the roots of their hosts, some rhizobia elicit the formation of specialized organs, called nodules, that they colonize intracellularly and within which they fix nitrogen to the benefit of the plant. Rhizobia do not form a homogenous taxon but are phylogenetically dispersed bacteria. How such diversity has emerged is a fascinating, but only partly documented, question. Although horizontal transfer of symbiotic plasmids or groups of genes has played a major role in the spreading of symbiosis, such gene transfer alone is usually unproductive because genetic or ecological barriers restrict evolution of symbiosis. Here, we experimentally evolved the usually phytopathogenic bacterium *Ralstonia solanacearum*, which was carrying a rhizobial symbiotic plasmid into legume-nodulating and -infecting symbionts. From resequencing the bacterial genomes, we showed that inactivation of a single regulatory gene allowed the transition from pathogenesis to legume symbiosis. Our findings indicate that following the initial transfer of symbiotic genes, subsequent genome adaptation under selection in the plant has been crucial for the evolution and diversification of rhizobia.

## Introduction

Bacteria known as rhizobia have evolved a mutualistic endosymbiosis of major ecological importance with legumes that contributes ca. 25% of global nitrogen cycling. Rhizobia induce the formation on legumes of root nodules that they colonize intracellularly [1] and in which they fix nitrogen to the benefit of the plant. Rhizobia are taxonomically, metabolically, and genetically diverse soil bacteria [2],[3]. They are currently distributed in 12 genera of α- and β-proteobacteria intermixed with saprophytes and pathogens. The occurrence of rhizobia in several distant genera is thought to have originated from repeated and independent events of horizontal transfer of key symbiotic functions in non symbiotic bacterial genomes [2],[4]. Symbiotic plasmid/island transfer has been proven both in the field and in the lab [5],[6]. However, horizontal gene transfer cannot solely account for the wide biodiversity of rhizobia, since only a few recipient bacteria—phylogenetically close to existing rhizobia [5]–[8]—turned into nitrogen-fixing legume symbionts. Which phylogenetic, genetic, or ecological barriers restrict evolution of symbiotic properties and how these barriers are overcome have not been investigated so far.

**Figure 1:**
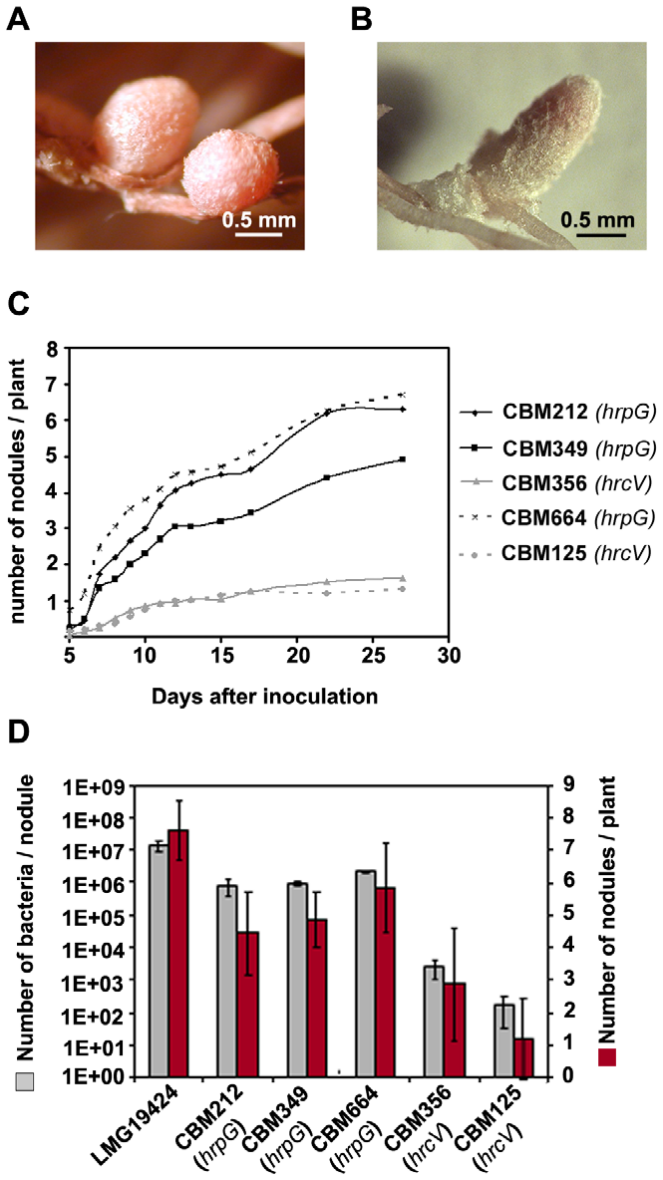
Nodulation of *M. pudica* by *C. taiwanensis* LMG19424, symbiotically evolved clones CBM356, CBM212, and CBM349, and mutant chimeric *Ralstonia* CBM125 and CBM664. (A) Nitrogen-fixing nodules formed by *C. taiwanensis* LMG19424. (B) Fix^−^ nodules formed by CBM212 on *M. pudica*. (C) Nodulation kinetics of the evolved clones and the mutants. (D) Number of nodules harvested at 14 days postinoculation and number of bacteria isolated per nodule. The number of in planta bacterial generations is estimated at 20 per nodule for CBM212 and CBM349 and 10 per nodule for CBM356.

Experimental evolution [9] coupled with genome resequencing [10] is a powerful approach to address the evolution of rhizobia. *Ralstonia solanacearum* and *Cupriavidus taiwanensis* are plant-associated β-proteobacteria with drastically different lifestyles. *R. solanacearum* is a typical root-infecting pathogen of over 200 host plant species. It intercellularly invades root tissues and heavily colonizes the vascular system, where excessive production of extracellular polysaccharides blocks water traffic, causing wilting [11],[12]. *Cupriavidus taiwanensis* is the major nitrogen-fixing symbiont of *Mimosa* spp. in Asia [13],[14] (see Figure 1A). Due to their phylogenetic and genomic distance (Figure S1), *C. taiwanensis* and *R. solanacearum* are ideally suited to act as symbiotic gene provider and recipient, respectively, in experimental evolution.

Here, we report on the experimental evolution of *R. solanacearum* carrying the symbiotic plasmid of *C. taiwanensis* into *Mimosa*-nodulating and -infecting symbionts. Two types of key adaptive mutations are described that are crucial for the transition from pathogenicity to mutualism. One allows nodulation to occur, whereas the other allows intracellular infection of plant cells, a very rare event in plant-associated bacteria.

## Results/Discussion

### Evolution of Symbiotically Proficient *R. solanacearum*

To generate our starting material, we transferred the 0.55-Mb symbiotic plasmid pRalta of *C. taiwanensis* LMG19424 into *R. solanacearum* strain GMI1000, generating the *Ralstonia* chimeric strain CBM124. pRalta carries nitrogen-fixation genes and a full complement of nodulation genes required for the synthesis of lipochitooligosaccharide Nod factors (NFs) [15] that trigger the plant developmental program of nodule organogenesis [16]. Nevertheless, CBM124 was unable to nodulate the *C. taiwanensis* legume host *Mimosa pudica* and retained the pathogenic properties of *R. solanacearum*, i.e., pathogenicity on *Arabidopsis thaliana* and hypersensitive response (HR) induction on tobacco (Figure S2). Note that *M. pudica* is not a host plant for *R. solanacearum*. Several lines of evidence indicated that CBM124 had a symbiotic potential that, for an unknown reason, could not be expressed. First, a *nodB*-*lacZ* transcriptional fusion was induced by the *nod*-inducer luteolin in a similar way in CBM124 and in *C. taiwanensis* (Table S1). Second, mass spectrometry analysis demonstrated that CBM124 produced NFs structurally identical to those of *C. taiwanensis* [15] (Figure S3). Third, CBM124 induced root hair proliferation and deformations on *M. pudica*, typical of those induced by NFs (see below), indicating that CBM124-produced NFs were active.

To isolate clones expressing symbiotic potential, we took advantage of specific traits of the rhizobium–legume symbiosis, (i) legume plants act as a trap by selecting rare, nodulation-proficient mutants in an otherwise non-nodulating population [17], (ii) a single bacterium enters and multiplies within the nodule [18], which implies that a rare nodulation-conferring mutation in a population is rapidly fixed, and (iii) nodulation, infection, and nitrogen fixation, are phenotypically clear-cut symbiotic stages. Both the original chimera CBM124 and a gentamicin-resistant derivative, CBM124GenR, were used to repeatedly inoculate sets of ca. 500 *M. pudica* seedlings grown in nitrogen-free conditions, as previously described [13]. Whereas no nodules were obtained using CBM124 as an inoculum, three nodules, which appeared 3–4 wk after inoculation, were recovered from three independent CBM124GenR inoculation experiments. One bacterial clone was isolated from each nodule, generating CBM212, CBM349, and CBM356. These three clones nodulated *M. pudica* with different kinetics and efficiencies (Figure 1). Their nodulation ability was, however, reduced relative to *C. taiwanensis* (Figure 1D and Figure S4), and all three clones were unable to fix nitrogen (Fix^−^).

### Identification of Key Adaptive Mutations for Symbiosis

We re-sequenced the three experimentally evolved clones as well as their immediate ancestor, CBM124GenR, using paired-end Illumina/Solexa sequencing technology (http://www.illumina.com/). Sequence data were mapped to the reference genome (6.37 Mb) based on the known genome sequences of *R. solanacearum* GMI1000 [19] and *C. taiwanensis* LMG19424 [15], and analyzed using the SNIPER software (S. Cruveiller and C. Medigue, unpublished data). We identified indels, SNPs (single nucleotide polymorphisms), and large deletions in the evolved clones relative to the CBM124GenR ancestor (Table S2). Among them, we focused on a large deletion as well as three SNPs that affected the HrpG-controlled virulence pathway in all three clones (Table 1). We confirmed the deletion and the SNPs by PCR amplification and Sanger resequencing.

**Table 1:** Validated SNPs affecting the HrpG-controlled virulence pathway and common deletion in all evolved clones.

Strain	Genes	Product	Mutation	Positions	Protein Modification
**CBM212**	Rsp0128 to Rsp0154		Deletion	148232–178934	
	*hrpG*	Response regulator	G>A	1083060	Q81^a^
**CBM349**	Rsp0128 to Rsp0154		Deletion	148232–178934	
	*hrpG*	Response regulator	G>A	1082676	Q209^a^
**CBM356**	Rsp0128 to Rsp0154		Deletion	148232–178934	
	*hrcV*	Type III secretion protein	G>A	1089958	Q589^a^

a Stop codon.

The ca. 33-kb deletion (Rsp0128–Rsp0154) of the *R. solanacearum* chromosome 2 removed 27 genes, including the *pme* gene coding for a pectin methylesterase involved in virulence and genes encoding a putative type II secretion system. This deletion was reconstructed in the chimera CBM124 by using the *cre-lox* system (see Material and Methods). The resulting strain did not nodulate *M. pudica*, indicating that this deletion either was not adaptive or alone could not account for nodulation. This region probably corresponds to an unstable region of the genome.

The regulatory protein HrpG controls the expression of many virulence determinants in *R. solanacearum* [20]. These include a type III secretion machinery (T3SS) and associate effector proteins that are regulated via the intermediate regulator *hrpB* [21] as well as a large ensemble of genes that are modulated by *hrpG* in an unidentified circuitry [20]. A stop mutation in the *hrcV* gene, which encodes a structural inner membrane protein at the base of the T3SS apparatus [22], was observed in CBM356, whereas both CBM212 and CBM349 harboured a stop mutation in the master regulator *hrpG* gene itself (Table 1). Consistently, all three clones exhibited a typical T3SS-defective phenotype, i.e., loss of HR induction on tobacco leaves (Figure S2). Although *C. taiwanensis* also possesses a T3SS of unknown function, it is not located on pRalta, thus ruling out the possibility that the impact on nodulation of the *R. solanacearum* virulence pathway was due to a modulation of indigenous *C. taiwanensis* T3SS. To assess the possible role of *hrcV* and *hrpG* gene inactivation in *M. pudica* nodulation, we inactivated the *hrcV* and *hrpG* genes in the original *Ralstonia* chimeric strain CBM124. Both CBM125 (*hrcV*) and CBM664 (Δ*hrpG*) were indeed found to nodulate *M. pudica*. Nonpolar disruption of *hrcS*, another T3SS structural gene, as well as independent *hrpG* inactivation by site-directed Tn5 mutation in the CBM124 background further confirmed the role of the T3SS and the *hrpG* gene in nodulation of *M. pudica*. The *hrcV* and *hrpG* mutants had symbiotic behaviours similar to that of the *hrcV* and *hrpG* evolved clones, respectively (Figure 1C and 1D).

### T3SS Inactivation Allows Chimeric *Ralstonia* to Nodulate and to Enter Root Hairs via Infection Threads

**Figure 2:**
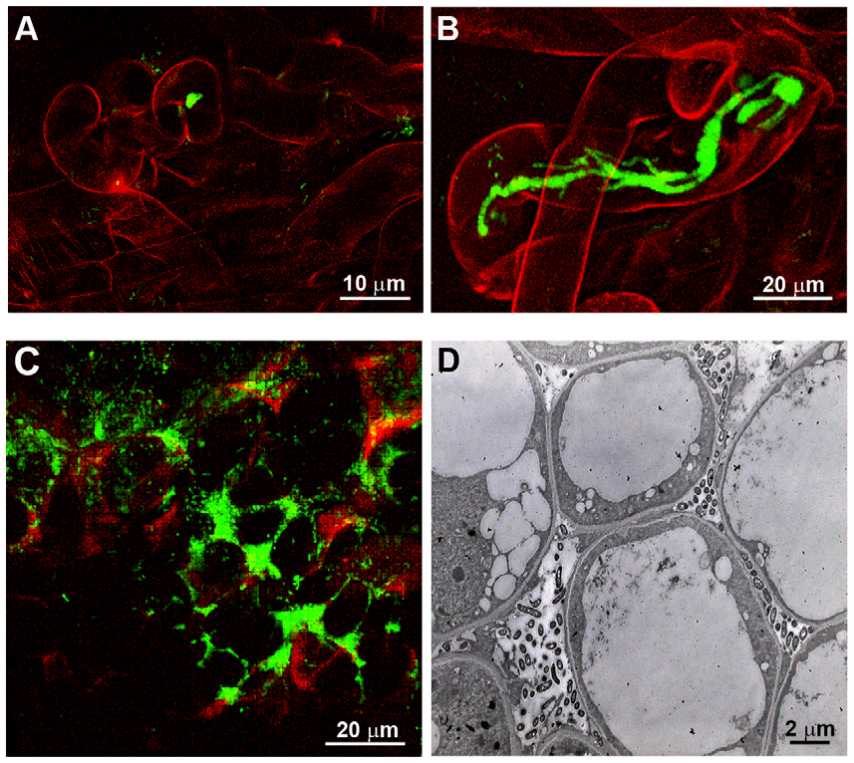
*hrcV* inactivation allows chimeric *Ralstonia* to nodulate and to enter root hairs via infection threads (ITs). (A) Inoculation with the chimeric strain CBM124-*gfp* resulted only in microcolony formation within curled hairs (no IT formation). (B–D), CBM125-*gfp* strains (*hrcV*) formed ITs in root hairs (B) and were located in intercellular spaces within nodules (C and D).

Like most rhizobia, *C. taiwanensis* invades *Mimosa* roots by means of transcellular infection threads (ITs), which are initiated from microcolonies entrapped within the curled root hairs known as shepherd's crooks [14] (Figure S5). Later on, ITs elongate into emerging nodules delivering bacteria into the plant cells. Each infected cell houses thousands of symbiosomes composed of internalized bacteria (called bacteroids) surrounded by plant-derived peribacteroid membranes. Mature *Mimosa* nodules induced by *C. taiwanensis* have the typical histology of indeterminate nodules, i.e., a single distal persistent meristem and peripheral vascular bundles (Figure S5). The ancestral chimeras CBM124 and CBM124GenR promoted root hair proliferation and deformations as well as shepherd's crooks. However, these chimeras showed a clear defect in IT initiation and elongation (Figure 2A and Figure S6). In contrast, well-elongated ITs were observed with the *hrcV* mutant CBM125 (Figure 2B). Nodules formed, which displayed the typical nodule structure (Figure S7), although often of irregular shape compared to those induced by *C. taiwanensis*. However the *hrcV* mutant only partially and extracellularly invaded the nodule (Figure 2C and 2D). A two-step inoculation experiment using differently labelled (*gfp* and *lacZ*) strains confirmed that extracellular bacteria inside nodules originated from ITs and did not result from intercellular penetration of bacteria from the nodule surface. A necrotic dark brown zone around which bacteria were distributed was often observed in the distal part of the infected zone of the nodules (Figure S7). Plant cell wall thickening next to extracellular bacteria was also suggestive of a plant structural defence response. This could act as a physical barrier to intracellular infection. In a similar way, the evolved clone CBM356 (*hrcV*) was able to form elongated ITs but did not permit invasion of nodule cells (Figure S7), strongly indicating that the *hrcV* mutation indeed accounted for the symbiotic phenotype of CBM356. We observed that a double mutant of the PopF1 and PopF2 translocons, which do not inhibit the formation of the T3SS apparatus in *R. solanacearum* but are required for protein effector injection in plant cells [23], had a similar phenotype (Figure S4), thus suggesting that a T3SS effector(s) is involved in blocking nodulation and early infection. Most interestingly, some rhizobia have been shown to use specialized host-targeting type III or type IV secretion systems to either extend or restrict legume host range (reviewed in [24]). Expression of these secretion systems is coordinated to nodulation gene expression. Effectors have been identified that can either be rhizobium specific or pathogen related. They have been proposed to modulate host (signalling) pathways, including plant-defence reactions triggered by the presence of infecting rhizobia [24]. Because *R. solanacearum* has more than 70 effectors [21], identification of the effector(s) responsible for blocking nodulation requires further work. Either nodulation is inhibited by effector-triggered immunity [25] or a T3SS effector(s) specifically interferes with the NF-signalling pathway.

### *hrpG* Inactivation Allows Intracellular Invasion of Nodule Cells

**Figure 3:**
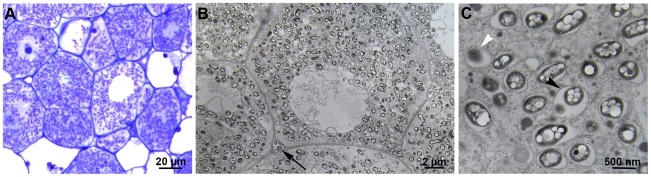
*hrpG* inactivation allows intracellular invasion of nodule cells. (A–C) CBM124Δ*hrpG* massively invaded plant cells intracellularly. A few bacteria were found in intercellular spaces ([B] arrow). Intracellular bacteria (bacteroids) were surrounded by a peribacteroid membrane ([C] black arrowhead) forming typical symbiosomes. Vesicles containing osmophile material ([C] white arrowhead) were often seen.

The *hrpG* mutant of CBM124 (CBM664), as well as the *hrpG* evolved clones CBM212 and CBM349, formed nodules on *M. pudica* that looked similar to those induced by *C. taiwanensis* (Figure S8). In young nodules, plant cells were massively intracellularly invaded (Figure 3A and 3B, and Figure S8), although the infected zone was restricted, compared to N_2_-fixing nodules formed by *C. taiwanensis*. Intracellular bacteria were surrounded by a peribacteroid membrane forming typical symbiosomes (Figure 3C). Nodules, however, showed early signs of degeneration generally 3 wk postinoculation, i.e., loss of cell-to-cell contact, cytoplasmic structure desegregation of nodule cells and degradation of the internalized bacteria (Figure S8). A few extracellular bacteria were found in nodules formed by the *hrpG* chimeric mutant and CBM212 and CBM349 clones (Figure 3B), which is never seen with *C. taiwanensis*. In these cases, no plant cell wall thickening could be observed in proximity to extracellular bacteria, suggesting that they did not induce plant defence reactions. To summarize, *hrpG* mutants and evolved clones were able to intracellularly invade nodule cells, contrary to *hrcV* mutants, although bacteroids were impaired for long-term maintenance. The regulatory gene *hrpG* thus controls one or several T3SS-independent functions interfering with plant cell entry. In plant-associated bacteria, massive intracellular infection is restricted to nodule bacteria. Hence, there is a paradox between the rarity of intracellular infection in plants and the ease with which this trait was acquired by a strictly extracellular pathogen. Mechanisms of plant cell entry in *C. taiwanensis* and in rhizobia in general are largely unknown, although it has been established that surface polysaccharides play a key role in host invasion [1]. Identification of the gene(s) downstream of *hrpG* controlling intracellular infection should shed light to this key, but still obscure, step of the symbiotic interaction.

### Conclusion

How rhizobia have emerged is a fascinating, but so far only partly documented, question. Although pioneering work 15 y ago established the role of lateral transfer in rhizobia evolution [5],[6], we and others [26],[27] have observed that in many instances, transfer of symbiotic loci did not increase symbiotic competence. Here, we show that a recipient genome—that is not immediately converted to a rhizobium upon transfer of a symbiotic plasmid—could rapidly evolve two specific symbiotic traits, i.e., nodulation and intracellular infection, under plant selection pressure. Although in our case, nitrogen fixation—and hence mutualism—was not achieved and evolved clones could be considered as cheaters [28], evolution of nodulation and infection capacities is the first step in the evolutionary process of reciprocal cooperation [29]. Extant rhizobial lineages diverged long before they acquired symbiotic properties [30], i.e., after legumes appeared on earth 60 million years ago. Our results show that adaptive genomic changes indeed allow effective dissemination of symbiotic traits over large phylogenetic and ecological distances. The fact that a single gene played a major role in the shift from extracellular pathogenesis to endosymbiosis reinforces previous reports that global regulators are preferred targets for evolution [31] and supports fluid boundaries between parasitism and mutualism.

Our knowledge of the rhizobium–legume symbiosis mainly comes from gene inactivation studies. Although a gain-of-function approach was first initiated ca. 25 y ago on *Agrobacterium* [8],[26],[32] and used thereafter [7],[33], the experimental evolution approach we describe here is novel, as it consists of the progressive and dynamic acquisition of symbiotic ability under plant selection pressure. Evolved clones gained symbiotic traits to different degrees, allowing for a future fine dissection of unexplored aspects of nodulation and intracellular infection. Serial in planta passages using the nodulating clones described here as ancestors should allow improvement of their symbiotic capacities, i.e., bacteroid maintenance and possibly nitrogen fixation. Other symbiotic stages, such as rhizosphere colonization, host specificity of nodulation, and nitrogen fixation, could similarly benefit from coupled experimental evolution and genome resequencing approaches.

## Materials and Methods

### Bacterial Strains, Plasmids, and Growth Conditions

Bacterial strains and plasmids used in this work are listed in Tables 2 and 3. *C. taiwanensis* strains were grown at 28°C on TY medium supplemented with 6 mM CaCl_2_ or quarter-strength minimal medium (MM) [34] supplemented with 10 mM disodium succinate and vitamin solution (1 µg/ml nicotinic acid, 1 µg/ml thiamine hydrochloride, 1 µg/ml pyridoxine hydrochloride, 100 µg/ml myo-inositol, 1 µg/ml calcium pantothenate, 1 µg/ml riboflavin, 1 µg/ml ascorbic acid, 1 µg/ml folic acid, 1 µg/ml cyanocobalamin, 1 µg/ml D-biotin). *R. solanacearum* strains were grown at 28°C on rich BG medium [35] or MM supplemented with 28 mM glucose. Antibiotics were used at the following concentrations (in micrograms per millilitre): streptomycin 600, spectinomycin 40, trimethoprim 100, tetracycline 10, gentamicin 25, chloramphenicol 50 for *E. coli* and 200 for *C. taiwanensis*, and kanamycin 50 for *E. coli*, and 30 for *R. solanacearum*.

**Table 2:** Strains used in this study.

Bacterium	Strain	Relevant Characteristics	Reference/Source
***C. taiwanensis***	LMG19424	Wild-type strain isolated from *Mimosa pudica* in Taiwan	[45]
	CBM832	LMG19424 derivative resistant to Str^R^	M. Hynes
	204	LMG19424-*gfp*	[14]
	CBM61	CBM832 pRalta::Tn5-B13S RP4-7, Str^R^, Chl^R^	This study
	CBM132	CBM832 pCBM01, Str^R^, Tet^R^	This study
***R. solanacearum***	GMI1000	Wild-type strain isolated from tomato in French Guyana	[35]
	GMI1425	GMI1000 *hrpG*::Tn5-B20, Kan^R^	[46]
	GMI1485	GMI1000::Tn5-B20-*lacZ*, Kan^R^	[12]
	GMI1596	GMI1000 *hrcS*::alpha-3, Kan^R^	[47]
	GMI1600	GMI1000-*gfp*, Kan^R^	[48]
	GMI1667	*popF1*::Ω, *popF2*::apra double mutant of GMI1000, Spe^R^, Str^R^, Gen^R^	[23]
	GMI1694	GMI1000 *hrcV*::Ω, Spe^R^, Str^R^	[49]
	GRS412	GMI1000 Rsp1236::pCZ367, Gen^R^	Christian Boucher
**Chimeric ** ***Ralstonia***	CBM124	GMI1000 pRalta::Tri, Tri^R^	This study
	CBM124GenR	CBM124 Rsp1236::pCZ367, Tri^R^, Gen^R^	This study
	CBM125	GMI1000 pRalta::Tri *hrcV*::Ω, Tri^R^, Spe^R^	This study
	CBM128	CBM124-*gfp*, Tri^R^, Kan^R^	This study
	CBM129	CBM125-*gfp*, Tri^R^, Kan^R^	This study
	CBM134	CBM124 pCBM01, Tri^R^, Tet^R^	This study
	CBM140	CBM124-*lacZ*, Tri^R^, Kan^R^	This study
	CBM141	CBM125-*lacZ*, Tri^R^, Kan^R^	This study
	CBM142	CBM124 *hrcS*::alpha-3, Tri^R^, Kan^R^	This study
	CBM145	CBM124 *popF1*::Ω, *popF2*::apra, Tri^R^, Spe^R^, Str^R^, Gen^R^	This study
	CBM212	Spontaneous nodulating clone isolated from *M. pudica* inoculated with CBM124GenR, Tri^R^, Gen^R^	This study
	CBM349	Spontaneous nodulating clone isolated from nod *M. pudica* inoculated with CBM124GenR, Tri^R^ Gen^R^	This study
	CBM351	CBM124 ΔRsp0126–Rsp0156::Kan, Tri^R^, Kan^R^	This study
	CBM356	Spontaneous nodulating clone isolated from *M. pudica* inoculated with CBM124GenR, Tri^R^, Gen^R^	This study
	CBM62	GMI1000 pRalta::Tn5-B13S, Tet^R^	This study
	CBM663	CBM124 *hrpG*::Tn5-B20, Tri^R^, Kan^R^	This study
	CBM664	CBM124 Δ*hrpG*::Kan, Tri^R^, Kan^R^	This study
	CBM703	CBM124GenR -*gfp*, Tri^R^, Kan^R^	This study

**Table 3:** Plasmids used in this study.

Plasmid	Relevant Characteristics	Reference/Source
p34E-Tp	Cassette vector with trimethoprim resistance gene, Tri^R^	[50]
pCBM01	pCZ388 containing 401 bp of the *nodB* promoter, Gen^R^, Tet^R^	This study
pCBM19	pCM184 containing Rsp0125 and Rsp0157 gene fragments, Amp^R^, Kan^R^, Tet^R^	This study
pCBM32	pCM184 containing *hrpG* upstream and downstream fragments, Amp^R^, Kan^R^, Tet^R^	This study
pCM184	*cre-lox* allelic exchange vector, Amp^R^, Kan^R^, Tet^R^	[51]
pCZ388	pLAFR6 derivative containing a promotorless *lacZ* gene, Gen^R^, Tet^R^	[521]
pMG02	pGEM-Teasy with a 2-kb fragment from pRalta carrying a Tri^R^ cassette	This study
pMH1801	pJQ18 derivative carrying a Tn5-B13S, *mob*, *sacRsacB*, Amp^R^, Chl^R^, Tet^R^, Kan^R^	[52]
RP4-7	Helper plasmid, Chl^R^	[54]

### Transfer of pRalta from *C. taiwanensis* to *R. solanacearum*

Transfer of pRalta to *R. solanacearum* was performed in three consecutive conjugation steps. Step 1. *C. taiwanensis* CBM832 was randomly transposon mutagenised using pMH1801 possessing the Tn5-B13S transposon which carries the mob site (oriT), an *npt*-*sacB*-*sacR* cassette and Tet-resistance. Step 2. Mutants were selected on TY supplemented with Tet and Str, and the helper plasmid, RP4-7, was individually introduced into each *C. taiwanensis* mutant. Step 3. *C. taiwanensis*::Tn5-B13S mutants carrying RP4-7 were then conjugated with *R. solanacearum*. Transconjugants were selected on MM supplemented with glucose and Tet. One Tn5-B13S mutagenised *C. taiwanensis* clone, CBM61, was successful in producing Tet-resistant *R. solanacearum* transconjugants. A selected transconjugant, CBM62, was verified as *R. solanacearum* containing pRalta by 16SrDNA and *nifH* gene amplification, and a seemingly intact pRalta was confirmed by a modified Eckhardt gel analysis [36]. The Tn5-B13S insertion in pRalta of CBM62 was found located within a putative transposase (see DNA Manipulation), and thus had not disrupted any gene essential for symbiosis, as confirmed by nodulation tests and microscopic observation of the mutagenised *C. taiwanensis* strain CBM61 used as donor for pRalta transfer. The Tn5-B13S, which contains *sacRsacB* genes that might interfere with plant tests, was exchanged in CBM62 with a trimethoprim (Tri) resistance cassette (see DNA Manipulation), giving rise to the *Ralstonia* chimeric strain GMI1000(pRalta::Tri), or CBM124.

The ancestral strain CBM124GenR was obtained by natural transformation [35] of CBM124 with genomic DNA from the *R. solanacearum* GRS412 strain (containing the Gen^R^ plasmid pCZ367 inserted in the Rsp1236 gene). Correct insertion of pCZ367 in CBM124GenR was verified by using a primer located upstream of the inactivated gene and a primer located in the *lacZ* gene of pCZ367. Transfer of pRalta::Tn5-B13S from CBM61 to *R. solanacearum* mutants was performed as indicated in step 3.

### Construction of Mutants

To construct CBM351, a CBM124 derivative deleted for the Rsp0128–Rsp0154 region, PCR fragments from the Rsp0125 and Rsp0157 genes (Rsp0126, Rsp0127, Rsp0155 and Rsp0156 are transposases) were amplified using oCBM494–oCBM495 and oCBM496–oCBM497 as primers and cloned into the EcoRI/NcoI and SacI/SacII restriction sites of pCM184, respectively. The modified plasmid was introduced into CBM124 by conjugation. Transconjugants resistant to kanamycin and sensitive to tetracycline were screened. The replacement of the Rsp0126–Rsp0156 region by the kanamycin resistance cassette in strain CBM351 was verified by PCR.

To construct CBM125, a *hrcV* mutant of CBM124, pRalta::Tn5-B13S, was transferred by conjugation from *C. taiwanensis* CBM61 to the *R. solanacearum hrcV* mutant GMI1694. The Tn5-B13S transposon was then replaced by the trimethoprim resistance cassette as described above.

To construct CBM142 and CBM145, the *hrcS* mutation and the *popF1 popF2* double mutation were introduced into CBM124 by natural transformation [35] of CBM124 with genomic DNA from the *R. solanacearum hrcS* mutant GMI1596 and the *popF1 popF2* double-mutant GMI1667, respectively. The presence of an inserted cassette in *hrcS*, *popF1*, and *popF2* was verified by PCR.

To construct the *hrpG* mutants, CBM663 and CBM664, two different methods were used. First the CBM124 strain was transformed with genomic DNA from *R. solanacearum hrpG*::Tn5-B20 mutant GMI1425. Transformants were selected on BG medium supplemented with trimethoprim and kanamycin. The Tn5-B20 insertion in *hrpG* was verified by PCR in strain CBM663. Second, PCR fragments upstream and downstream from *hrpG* were amplified using oCBM622–oCBM623 and oCBM624–oCBM625 as primers and cloned into the EcoRI/KpnI and SacII/HpaI restriction sites of pCM184, respectively. The resulting plasmid was introduced into CBM124 by conjugation. Transconjugants resistant to kanamycin and sensitive to tetracycline were screened. The replacement of *hrpG* by the kanamycin resistance cassette was verified by PCR in strain CBM664.

### DNA Manipulation

Primers used for DNA amplification are listed in Table S3.

To determine the precise location of the Tn5-B13S insertion point in pRalta of CBM61 and CBM62, tail-PCR was performed with arbitrary primer AD1 or AD4 [37] in combination with three sequential Tn5-specific primers designed from the terminal arms of the Tn5 transposon, oCBM183, oCBM184, and oCBM185.

For Tn5-B13S insertion exchange by Tri^R^ cassette, a 2-kb PCR fragment, corresponding to approximately 1 kb each side of the Tn5-B13S insertion point, was amplified from LMG19424 using primers oCBM196 and oCBM198 and cloned into pGEM-Teasy (Promega). The Tri^R^ cassette isolated from p34E-Tp digested by BamHI was then introduced in the BglII site of the fragment, generating pMG02. This BglII site was located only 6 bp from the Tn5-B13S insertion point in CBM62. ScaI linearized pMG02 DNA was used to transform naturally competent *R. solanacearum* chimeric strains containing pRalta::Tn5-B13S. The exchange of Tn5-B13S with the trimethoprim cassette was verified by establishing that the strain had lost resistance to tetracycline and could grow on 5% sucrose.

For the construction of pCBM01, the promoter region of *nodB* was amplified using oCBM203 and oCBM211 as primers and cloned into pGEM-Teasy (Promega), cleaved from pGEM-Teasy with HindIII and PstI, and then directionally cloned into the same sites of the *lacZ* transcriptional fusion in pCZ388. pCBM01 was introduced in *C. taiwanensis* and *R. solanacearum* strains by conjugation.

The *lacZ*- and *gfp*-derived strains were obtained by natural transformation with genomic DNA from strains GMI1485 and GMI1600, respectively.

### Solexa Re-Sequencing and Mutation Analysis

Sequence data production was performed by the C.E.A/IG/Genoscope (Evry). Paired-end libraries were prepared following the protocol recommended by Illumina Inc. (http://www.illumina.com). For each strain, more than 5 million paired-end reads (*L* = 72 bp = 2×36 bp) were generated with Genome Analyzer sequencing system, leading to a ca. 60× total coverage of the reference genome (Table S4). Taking advantage of the local production of raw sequencing data, a bioinformatic pipeline called SNiPer (S. Cruveiller and C. Medigue, unpublished data) and based on ssaha2 alignment software (Sequence Search and Alignment by Hashing Algorithm [38] has been implemented. This pipeline allows the detection of small variations (SNPs and InDels) between a collection of short reads and a reference sequence, this latter being either a consensus produced by assemblers or a previously published one.

SNiPer is a shell script that automatically sets the alignments parameters depending on the kind of reads (ABI-3730/454-GSFLX/Solexa/SOLiD) being used, launches the various parts of the detection pipeline, and controls for all tasks having been completed without errors. The detection of SNPs and indels is achieved in four main steps: (1) The data preparation, which consists in (i) the conversion of sequencing raw data (i.e., reads files) into Sanger Institute FastQ formatted files; (ii) the removal of duplicated reads (quite common when using Solexa platform) so as to keep exactly one copy of each read; and (iii) the split of paired-ends reads into single-end reads when required. (2) Reads mapping onto a reference molecule using the ssaha2 package [38]. This package combines the SSAHA searching algorithm (sequence information is encoded in a perfect hash function) aiming at identifying regions of high similarity, and the cross_match sequence alignment program (http://www.phrap.org/phredphrapconsed.html), which aligns these regions afterwards using a banded Smith-Waterman-Gotoh algorithm [39],[40]. (3) Based on the characteristics of reads alignments onto the reference molecule, a file containing the lists of all possible events is generated. (4) Each event is then scored so as to keep only significant ones. This score takes into account the reference base coverage (i.e., the number of reads mapping a given location) and the quality of bases of reads displaying a change at that particular location as well.

The ca. 5 million paired-end reads were split into single reads and mapped on the reference genome (the two replicons of *Ralstonia solanacearum* GMI1000 [RefSeq acc. NC_003295.fna and NC_003296.fna for the chromosome and the megaplasmid respectively]+the nodulation plasmid of *Cupriavidus taiwanensis* LMG19424 [RefSeq acc. NC_010529.fna]) using SNiPer. Among the 10 million single reads, around 7 millions were successfully mapped, leading to an effective coverage of the three reference molecules higher than 30× (Table S4), hence warranting a reliable detection of changes. The remaining unmapped reads (3 million on average) correspond to reads that could neither be mapped unambiguously (i.e., repeat regions, insertion sequences, rDNA, etc.) nor be mapped at all (i.e., fragment of sequences not present in the references).

### Plant Assays and Cytological studies

Pathogenicity assays with *M. pudica* and *Arabidopsis thaliana* ecotype Col-0 were performed according to Deslandes et al. [41]. Root inoculations used the method of cutting 2 cm from the bottom of Jiffy pot–grown plants, followed by immersion for 5 min in a suspension of bacteria grown overnight and diluted to an OD_600_ of 0.1 in water. *R. solanacearum* and derivatives were tested for the HR ability by infiltrating a bacterial culture adjusted to 10^8^ cells/millilitre into tobacco (cultivar Bottom Special) leaf parenchyma as described previously [35].

For *M. pudica* nodulation assay and cytology, seeds were surface sterilised and planted under sterile conditions using the tube method of Gibson as previously described [13], (except tubes contained Fahraeus [42] slant agar and liquid water). For the selection of nodulating evolved clones, 10^7^ bacteria par tube were used as inoculum. Otherwise, 10^4^ bacteria were routinely inoculated per tube unless specified. Nitrogen fixation was estimated by visual observation of the vigour and foliage colour of 40/60-d-old plants on at least 20 plants. For reisolation of nodule bacteria, nodules were surface sterilised 10 min with 2.6% sodium hypochlorite, rinsed five times, then crushed and dilutions plated on the appropriate solid medium. For each *M. pudica* tube, ex planta number of bacterial generations is estimated at a maximum of 5, and in planta generation number is calculated using the formula log(number of bacteria/nodule)/log_2_.

*LacZ*-tagged infecting bacteria were stained according to the standard procedure. Briefly, roots were fixed in glutaraldehyde 1.5% in K phosphate buffer for 30 min under vacuum condition followed by 1 h at room temperature. After washing, roots were incubated overnight with the staining solution at 28°C (0.1 M K phosphate [pH 7.4], 2 mM K ferricyanide, 2 mM K ferrocyanide, and 0.08% of X-gal in dimethylformamide). Roots were washed and used for microscopic analysis. To analyse infection of *gfp*-tagged bacteria, root and nodules were fixed in paraformaldehyde 3.7% in phosphate buffered saline (PBS) for 30 min under vacuum, then washed and used directly or cut for nodule sections 60-µm thick using a Leica VT1000S vibratome. Samples were observed by using a fluorescence (Zeiss Axiophot Fluorescence microscope) or confocal microscope (Leica SP2).

For fine histological examination, nodules were fixed in glutaraldehyde (2.5% in phosphate buffer 0.1 M [pH 7.4]), osmium treated, dehydrated in an alcohol series, and embedded in Epon 812. Semithin nodule sections were observed by brightfield microscopy after staining in 0.1% aqueous toluidine blue solution and observed under a Zeiss Axiophot light microscope. Ultrathin sections were stained with uranyl acetate and observed with a Hitachi EM600 electron microscope.

For the two-step infections, we proceeded as follows. *M. pudica* plants, grown as described above, were first infected with the *lacZ*-tagged *hrcV* chimeric strain. After 9 d of infection, once nodules were formed, a secondary infection was performed by using the *gfp*-tagged *hrcV* chimeric strain. Two weeks after, nodules were fixed in paraformaldehyde 3.7% as previously described and used for cytological analysis.

### β-Galactosidase Assays

Strains were grown overnight at 28°C in MM supplemented with the appropriate carbon source, vitamins, and tetracycline. Overnight cultures were then diluted to an OD_600_ of 0.005–0.01 in MM with tetracycline ±15 µM final concentration of luteolin and grown a minimum of 16 h until an OD_600_ of 0.7 was reached. The cultures were then assayed for β-galactosidase activity (Miller units) according to Miller, 1972 [43]. The β-galactosidase activities represent an average of quadruplicate samples from two separate experiments.

### Nod Factor Purification and Characterization

NFs were produced, purified, and characterized as previously described [15].

## Supporting Information

Figure S1 **Phylogenetic and genomic relationships between** ***C. taiwanensis*** **and** ***R. solanacearum*** **.** (A) Rooted 16S rDNA tree of *Cupriavidus* and *Ralstonia* species. The scale bar represents 5% of sequence divergence. Adapted from [44]. (B) Genome organization of *C. taiwanensis* LMG19424 and *R. solanacearum* GMI1000. (C) Synteny plots between *C. taiwanensis* LMG19424 and *R. solanacearum* GMI1000 genomes. The line plots have been obtained using synteny results between chromosomes 1 as well as chromosomes 2 of both genomes. Synteny groups containing a minimum of three genes are drawn in green for colinear regions, and in red for inverted regions. The display has been obtained using the MaGe graphical interface of the CupriaviduScope project (https://www.genoscope.cns.fr/agc/mage).
(0.19 MB PPT)

Figure S2 **Hypersensitive response elicited on the nonhost plant** ***Nicotiana tabacum*** **.** The tobacco leaf was infiltrated with a 10^8^ colony-forming units/millilitre suspension of *R. solanacearum* derivative strains. GMI1000, wild-type *R. solanacearum*. CBM124GenR, ancestral chimeric *Ralstonia*. CBM212, CBM349, CBM356, *Mimosa*-nodulating evolved clones. CBM125, *hrcV* chimera. The photograph was taken 48 h after infiltration.
(2.49 MB TIF)

Figure S3 **Compared structures of Nod factors from** ***C. taiwanensis*** **and chimeric CBM124.** Electrospray ionisation-mass spectrometry (ESI-MS) spectrum in the negative ionisation mode of high-performance liquid chromatography fractions eluting at 36% AcCN in water obtained from LMG19424 (A), and CBM124 (B). Molecular ions [M-H]^−^ at mass-to-charge ratio (m/z) 1391.8 correspond to an oligomer of five glucosamine units, substituted by a vaccenic acid (C_18∶1_), a methyl, a carbamoyl, and a sulphate group. Species at m/z 1365.7 and at m/z 1348 correspond to the same basic structure with a palmitic acid (C_16∶0_) instead of the vaccenic acid with or without the carbamoyl group, respectively.
(0.66 MB TIF)

Figure S4 **Compared nodulation of** ***M. pudica*** **by** ***C. taiwanensis*** **LMG19424 and the evolved clone CBM212 (A), and by the** ***hrcV*** **and** ***popF1popF2*** **mutants of the chimeric** ***Ralstonia*** **(B).** Plants were grown in Gibson tubes containing Fahraeus slant agar and 0.25× liquid Jensen. At least 20 plantlets were inoculated (10^7^ bacteria per tube) per strain.
(0.76 MB TIF)

Figure S5 **Nodulation and infection of** ***M. pudica*** **by** ***C. taiwanensis*** **.** (A) Root hair deformation following *C. taiwanensis* inoculation. (B and C) Infection threads of green *gfp*-tagged bacteria growing from infection sites with especially pronounced examples of branched and multiple infection threads (C). (D) Young nodules. (E and F) Nodule sections showing cells infected with *gfp*-tagged (E) or bacteria stained with toluidine blue (F). (G and H) Intracellular invasion of vegetal cells. Note the absence of bacteria in intercellular spaces. (I) Intracellular bacteria (bacteroids) surrounded by a peribacteroid membrane (arrow) forming typical symbiosomes.
(7.37 MB TIF)

Figure S6 **Infection of** ***M. pudica*** **by ancestral chimeric** ***Ralstonia*** **CBM124 (A, C, and D) and CBM124GenR (B).** (A) Root hair deformation following inoculation. (B and C) Microcolony of green *gfp*-tagged bacteria in curled root hair structures, and abortive ITs ([C] white arrow). (D) Dead root hair completely filled with blue *lacZ*-tagged bacteria, occasionally observed.
(2.78 MB TIF)

Figure S7 **Nodulation and extracellular infection of** ***M. pudica by the hrcV*** **chimeric mutant CBM125 (A–F and H) and the evolved clone CBM356 (G and I–K).** (A) Root hair deformation. (B and C) Formation of infection threads from infection sites within curled root hairs. ITs were fewer and delayed as compared to *C. taiwanensis*. Note they were also less branched and thicker. (D) Nodule of irregular shape. (E) Blue coloration indicating the presence of *lacZ*-tagged bacteria in limited infected zone of the nodule. (F) Nodule section showing vascular bundles (arrow) and a necrotic zone surrounded by bacteria tagged with GFP (arrowhead). (G) Nodule section showing intercellular spaces filled with bacteria (arrow). (H–K) Electronic microscopy observation of intercellular bacteria and cell wall thickening (asterisks) (H, J, K), and ITs (I).
(7.51 MB TIF)

Figure S8 **Nodulation and intracellular infection of** ***M. pudica*** **by** ***hrpG*** **chimeric mutant CBM664 (A and C) and evolved clones CBM212 (B, D, and F–K) and CBM349 (E).** (A and B) Young nodules. (C and D) Nodule sections showing the infected zone. (E–G) Massive intracellular invasion in nodules. (G) Note the presence of bacteria in intercellular spaces. (H) Intracellular bacteria surrounded by a peribacteroid membrane forming typical symbiosomes (arrow). Osmophile material containing vesicles (arrowhead), probably involved in premature symbiosome degradation, were often associated with symbiosomes. PHB (Polyhydroxybutyrate) storage granules were present in bacteria (asterisks). (I) Infection pocket within intercellular space. (J and K) Premature senescence of 5-wk-old nodules with cytoplasmic structure desegregation of vegetal cells, loss of cell-to-cell contact, and numerous empty symbiosomes (arrow).
(9.12 MB TIF)

Table S1 **Expression of a nodB::lacZ fusion in** ***C. taiwanensis*** **and chimeric** ***Ralstonia*** **in response to luteolin 15 µM.**
(0.03 MB DOC)

Table S2 **Number of mutations in evolved clones relative to the immediate ancestor CBM124GenR.**
(0.03 MB DOC)

Table S3 **List of primers.**
(0.06 MB DOC)

Table S4 **Characteristics of raw sequencing data output by the Illumina Genome Analyzer^a^ and SNiPer primary results^b^ for the strains under study.**
(0.03 MB DOC)

## Abbreviations

HR: hypersensitive response
IT: infection thread
NF: Nod factor
T3SS: type III secretion system
