# Supplementary material for: Experimental Evolution of a Plant Pathogen into a Legume Symbiont
Source: PLoS Biol. 2010 Jan 12;8(1):e1000280. doi: 10.1371/journal.pbio.1000280 (PMC2796954; doi:10.1371/journal.pbio.1000280)
Supplement: Figure S1 — Phylogenetic and genomic relationships between C. taiwanensis and R. solanacearum . (A) Rooted 16S rDNA tree of Cupriavidus and Ralstonia species. The scale bar represents 5% of sequence divergence. Adapted from [44]. (B) Genome organization of C. taiwanensis LMG19424 and R. solanacearum GMI1000. (C) Synteny plots between C. taiwanensis LMG19424 and R. solanacearum GMI1000 genomes. The line plots have been obtained using synteny results between chromosomes 1 as well as chromosomes 2 of both genomes. Synteny groups containing a minimum of three genes are drawn in green for colinear regions, and in red for inverted regions. The display has been obtained using the MaGe graphical interface of the CupriaviduScope project (https://www.genoscope.cns.fr/agc/mage). (0.19 MB PPT) [file pbio.1000280.s001.ppt]

## Slide 1
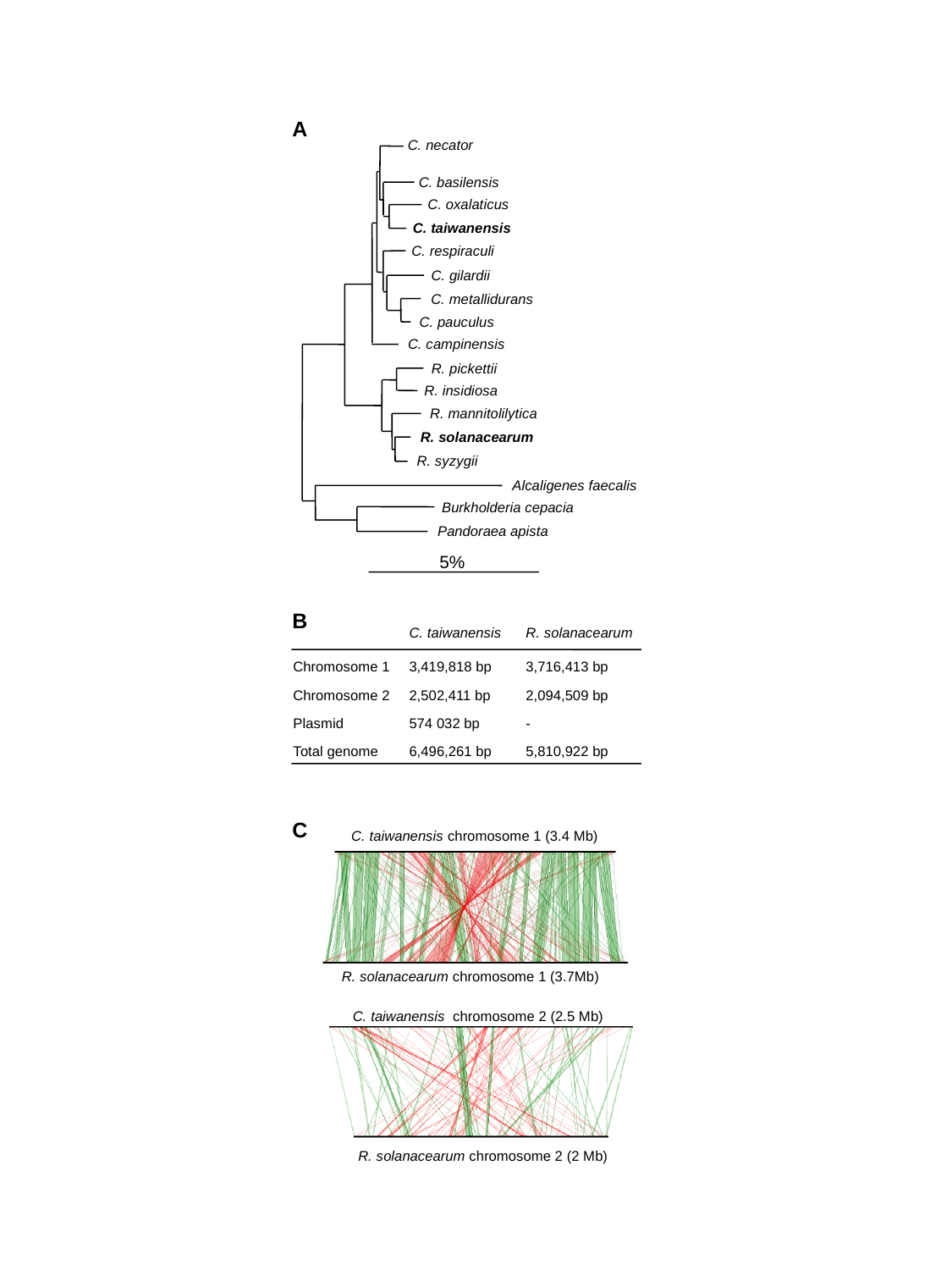

A
C. necator
C. basilensis
C. oxalaticus
C. taiwanensis
C. respiraculi
C. gilardii
C. metallidurans
C. pauculus
C. campinensis
R. pickettii
R. insidiosa
R. mannitolilytica
R. solanacearum
R. syzygii
Alcaligenes faecalis
Burkholderia cepacia
Pandoraea apista
5%
B
C. taiwanensis
R. solanacearum
Chromosome 1
3,419,818 bp
3,716,413 bp
Chromosome 2
2,502,411 bp
2,094,509 bp
Plasmid
574 032 bp
-
Total genome
6,496,261 bp
5,810,922 bp
C
C. taiwanensis chromosome 1 (3.4 Mb)
R. solanacearum chromosome 1 (3.7Mb)
C. taiwanensis chromosome 2 (2.5 Mb)
R. solanacearum chromosome 2 (2 Mb)
