# Supplementary figures and images for: Experimental Evolution of a Plant Pathogen into a Legume Symbiont
Source: PLoS Biol. 2010 Jan 12;8(1):e1000280. doi: 10.1371/journal.pbio.1000280 (PMC2796954; doi:10.1371/journal.pbio.1000280)

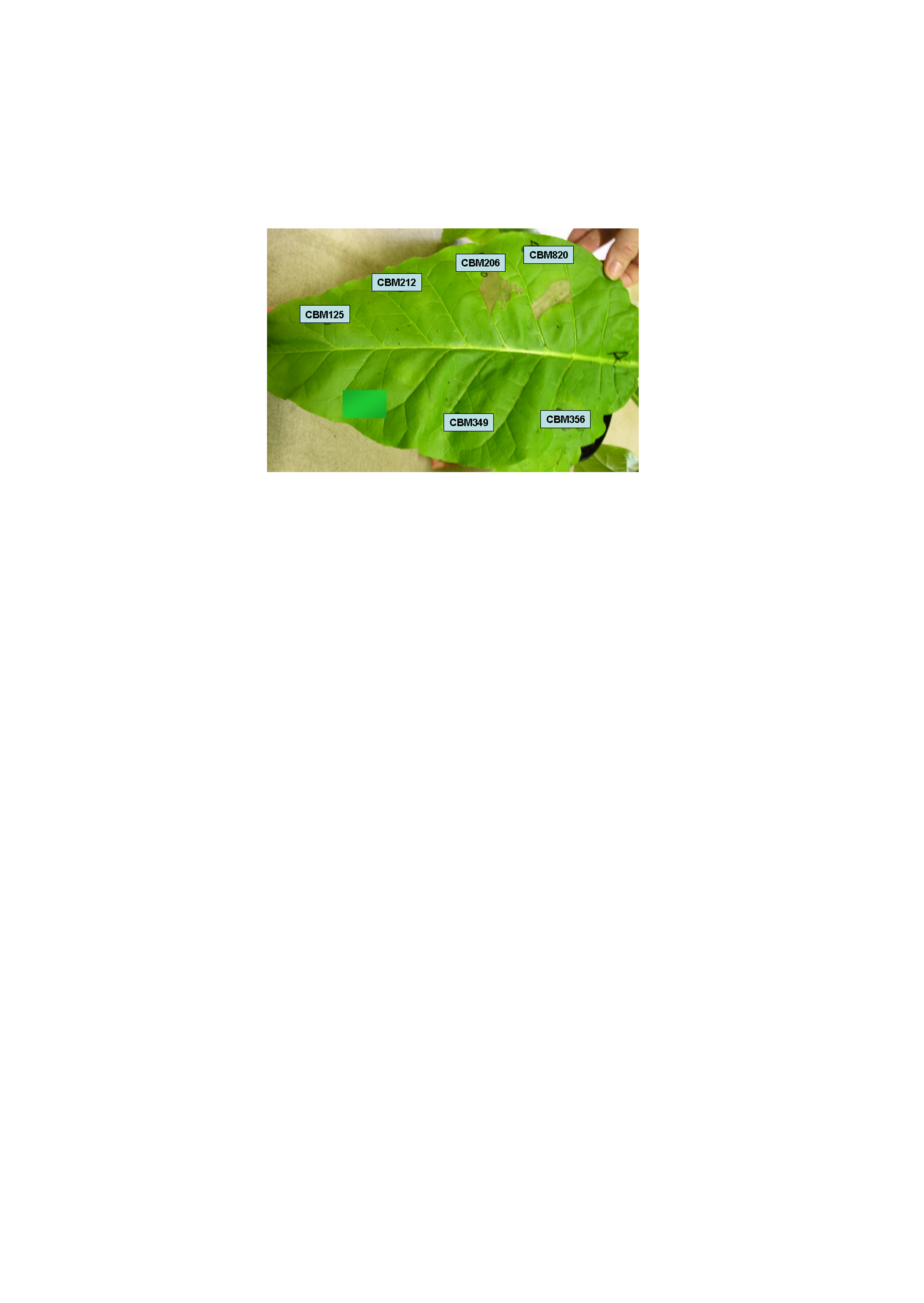

Supplement: Figure S2 — Hypersensitive response elicited on the nonhost plant Nicotiana tabacum . The tobacco leaf was infiltrated with a 108 colony-forming units/millilitre suspension of R. solanacearum derivative strains. GMI1000, wild-type R. solanacearum. CBM124GenR, ancestral chimeric Ralstonia. CBM212, CBM349, CBM356, Mimosa-nodulating evolved clones. CBM125, hrcV chimera. The photograph was taken 48 h after infiltration. (2.49 MB TIF) [file pbio.1000280.s002.tif]

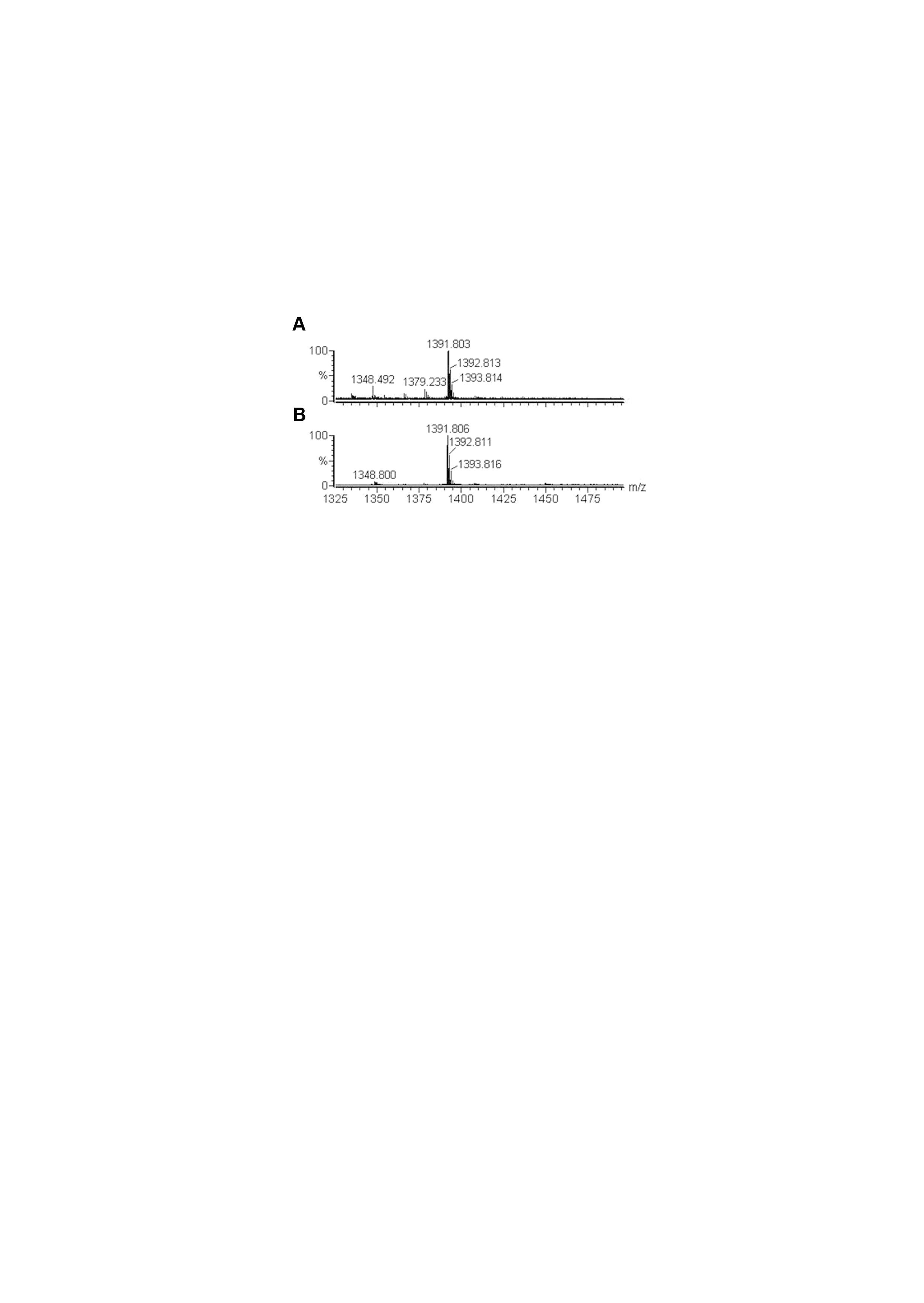

Supplement: Figure S3 — Compared structures of Nod factors from C. taiwanensis and chimeric CBM124. Electrospray ionisation-mass spectrometry (ESI-MS) spectrum in the negative ionisation mode of high-performance liquid chromatography fractions eluting at 36% AcCN in water obtained from LMG19424 (A), and CBM124 (B). Molecular ions [M-H]− at mass-to-charge ratio (m/z) 1391.8 correspond to an oligomer of five glucosamine units, substituted by a vaccenic acid (C18∶1), a methyl, a carbamoyl, and a sulphate group. Species at m/z 1365.7 and at m/z 1348 correspond to the same basic structure with a palmitic acid (C16∶0) instead of the vaccenic acid with or without the carbamoyl group, respectively. (0.66 MB TIF) [file pbio.1000280.s003.tif]

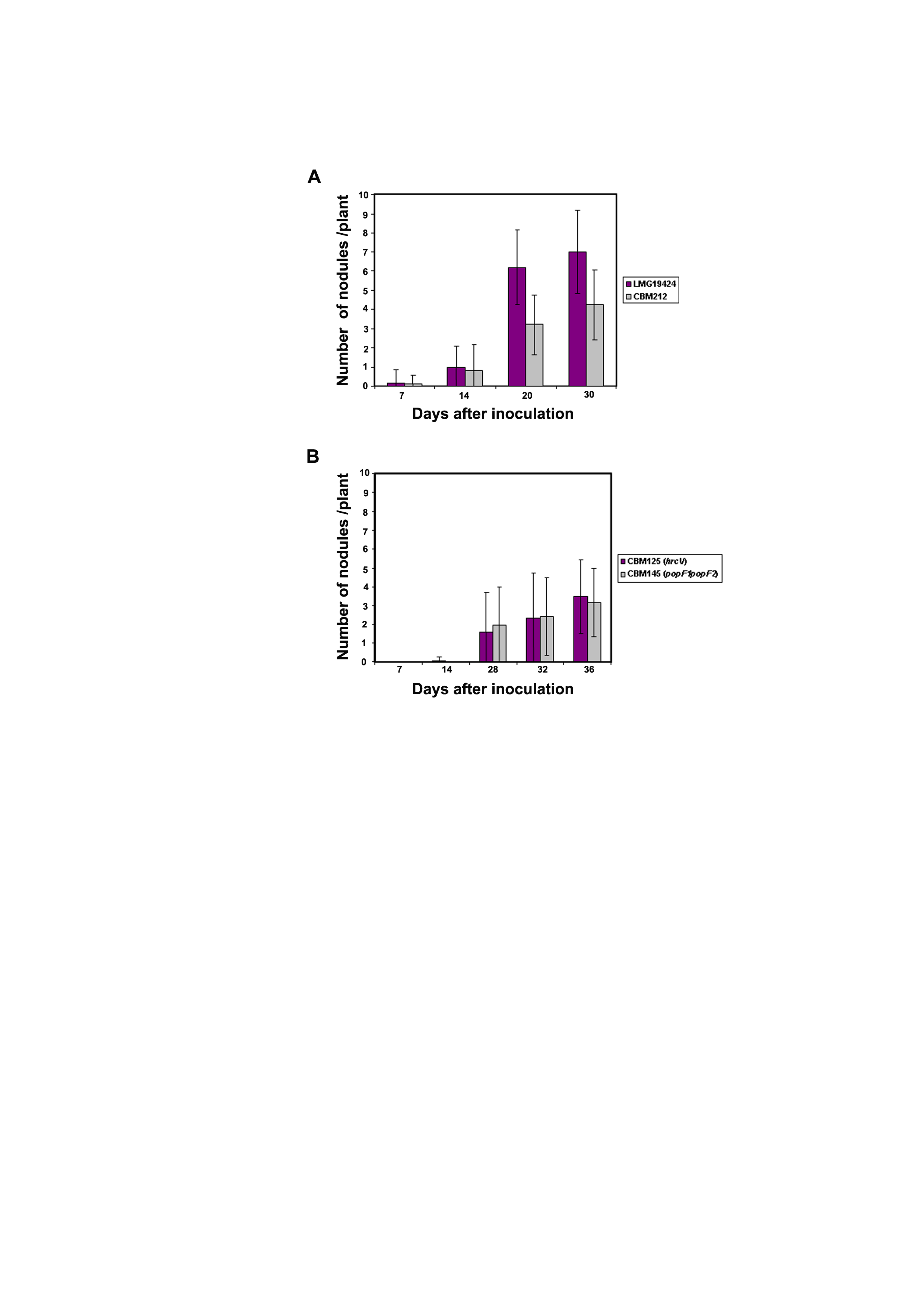

Supplement: Figure S4 — Compared nodulation of M. pudica by C. taiwanensis LMG19424 and the evolved clone CBM212 (A), and by the hrcV and popF1popF2 mutants of the chimeric Ralstonia (B). Plants were grown in Gibson tubes containing Fahraeus slant agar and 0.25× liquid Jensen. At least 20 plantlets were inoculated (107 bacteria per tube) per strain. (0.76 MB TIF) [file pbio.1000280.s004.tif]

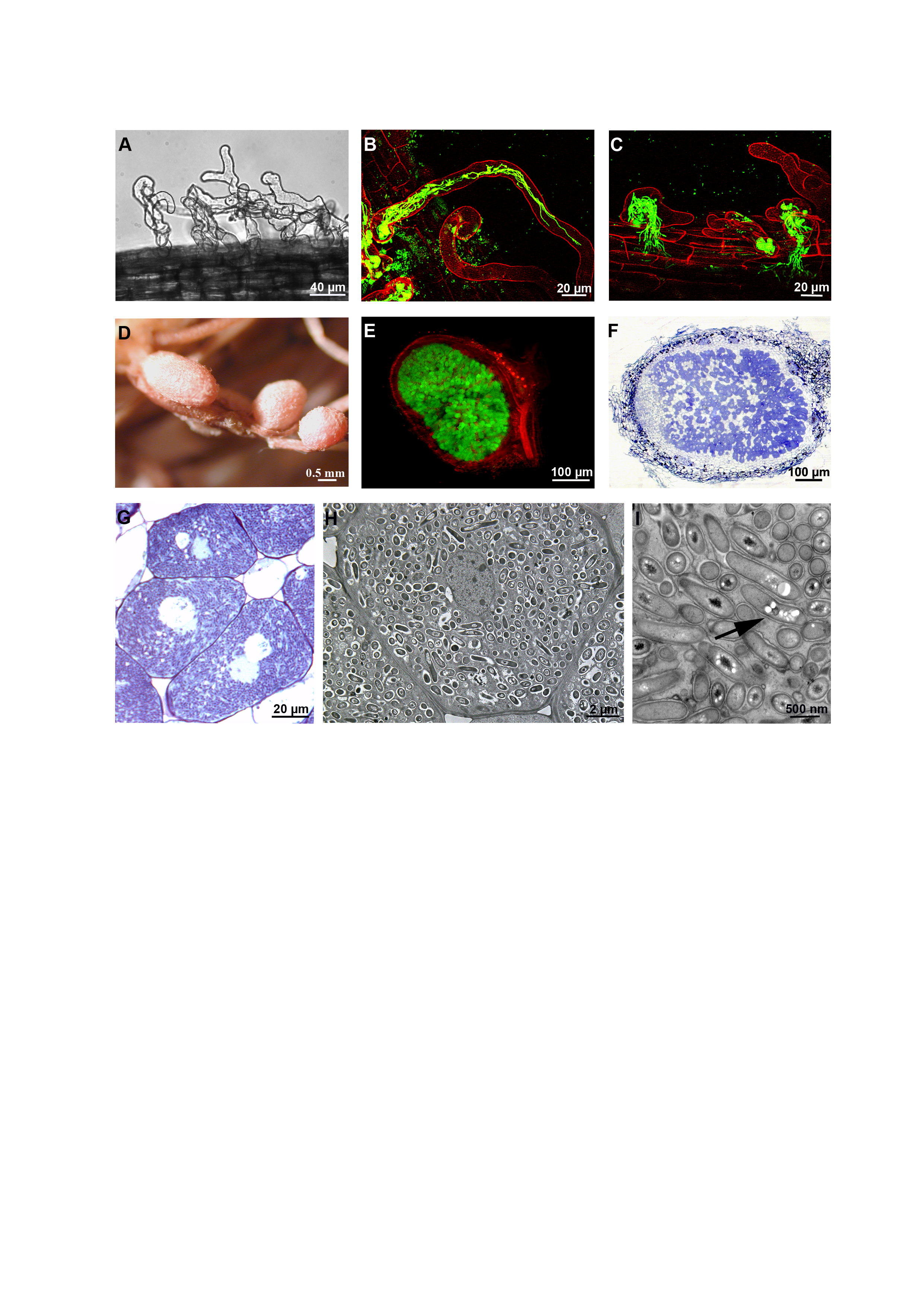

Supplement: Figure S5 — Nodulation and infection of M. pudica by C. taiwanensis . (A) Root hair deformation following C. taiwanensis inoculation. (B and C) Infection threads of green gfp-tagged bacteria growing from infection sites with especially pronounced examples of branched and multiple infection threads (C). (D) Young nodules. (E and F) Nodule sections showing cells infected with gfp-tagged (E) or bacteria stained with toluidine blue (F). (G and H) Intracellular invasion of vegetal cells. Note the absence of bacteria in intercellular spaces. (I) Intracellular bacteria (bacteroids) surrounded by a peribacteroid membrane (arrow) forming typical symbiosomes. (7.37 MB TIF) [file pbio.1000280.s005.tif]

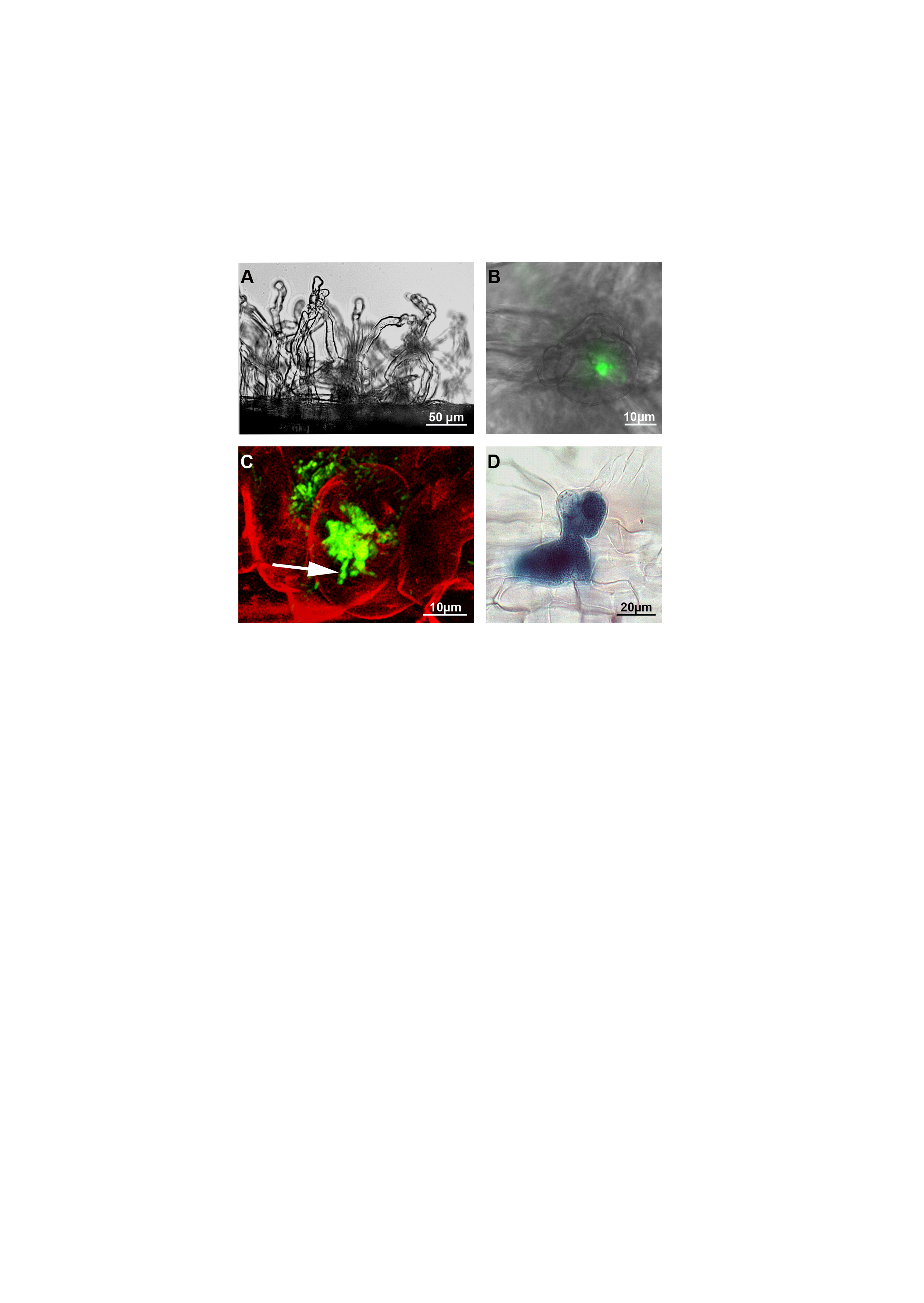

Supplement: Figure S6 — Infection of M. pudica by ancestral chimeric Ralstonia CBM124 (A, C, and D) and CBM124GenR (B). (A) Root hair deformation following inoculation. (B and C) Microcolony of green gfp-tagged bacteria in curled root hair structures, and abortive ITs ([C] white arrow). (D) Dead root hair completely filled with blue lacZ-tagged bacteria, occasionally observed. (2.78 MB TIF) [file pbio.1000280.s006.tif]

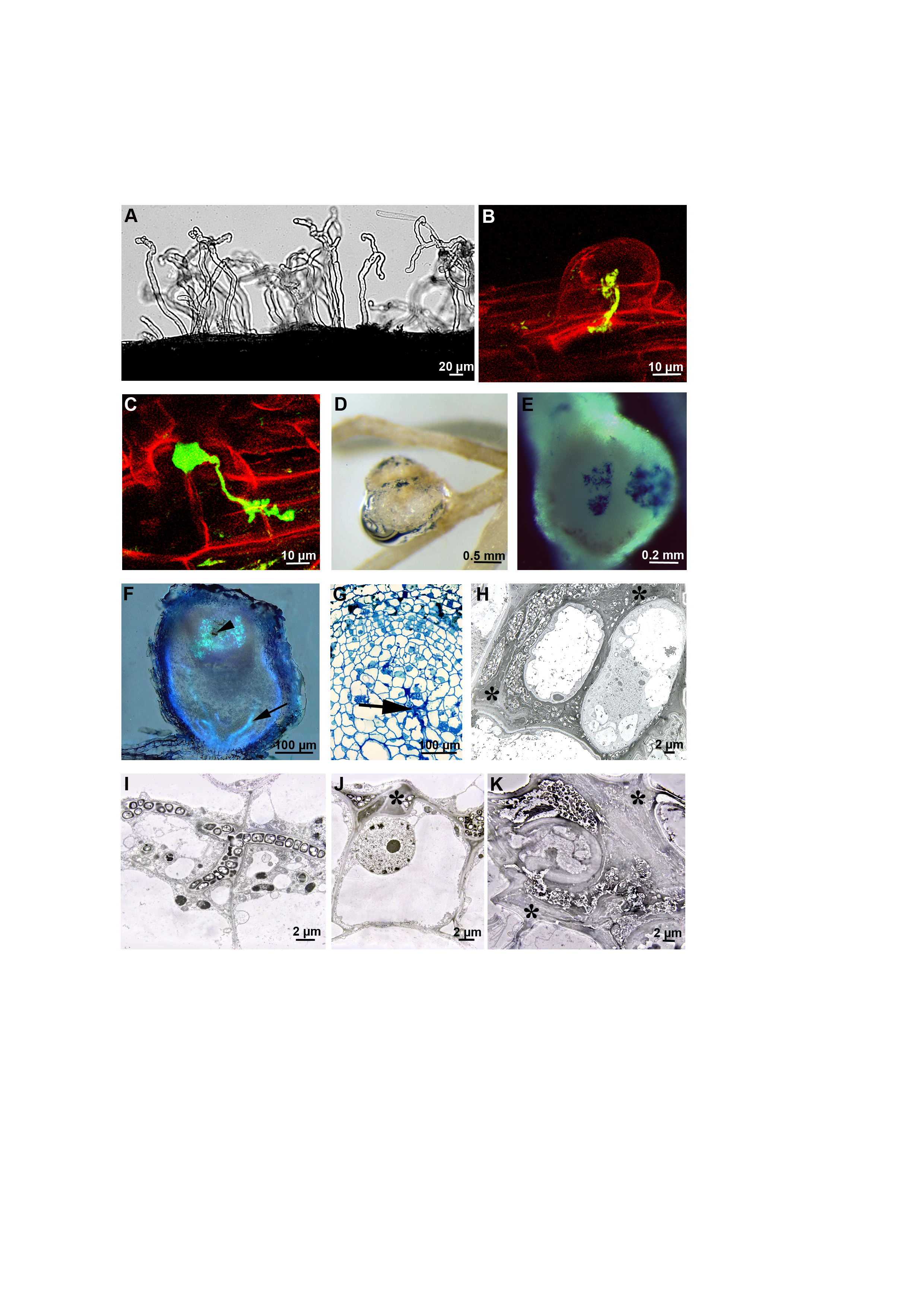

Supplement: Figure S7 — Nodulation and extracellular infection of M. pudica by the hrcV chimeric mutant CBM125 (A–F and H) and the evolved clone CBM356 (G and I–K). (A) Root hair deformation. (B and C) Formation of infection threads from infection sites within curled root hairs. ITs were fewer and delayed as compared to C. taiwanensis. Note they were also less branched and thicker. (D) Nodule of irregular shape. (E) Blue coloration indicating the presence of lacZ-tagged bacteria in limited infected zone of the nodule. (F) Nodule section showing vascular bundles (arrow) and a necrotic zone surrounded by bacteria tagged with GFP (arrowhead). (G) Nodule section showing intercellular spaces filled with bacteria (arrow). (H–K) Electronic microscopy observation of intercellular bacteria and cell wall thickening (asterisks) (H, J, K), and ITs (I). (7.51 MB TIF) [file pbio.1000280.s007.tif]

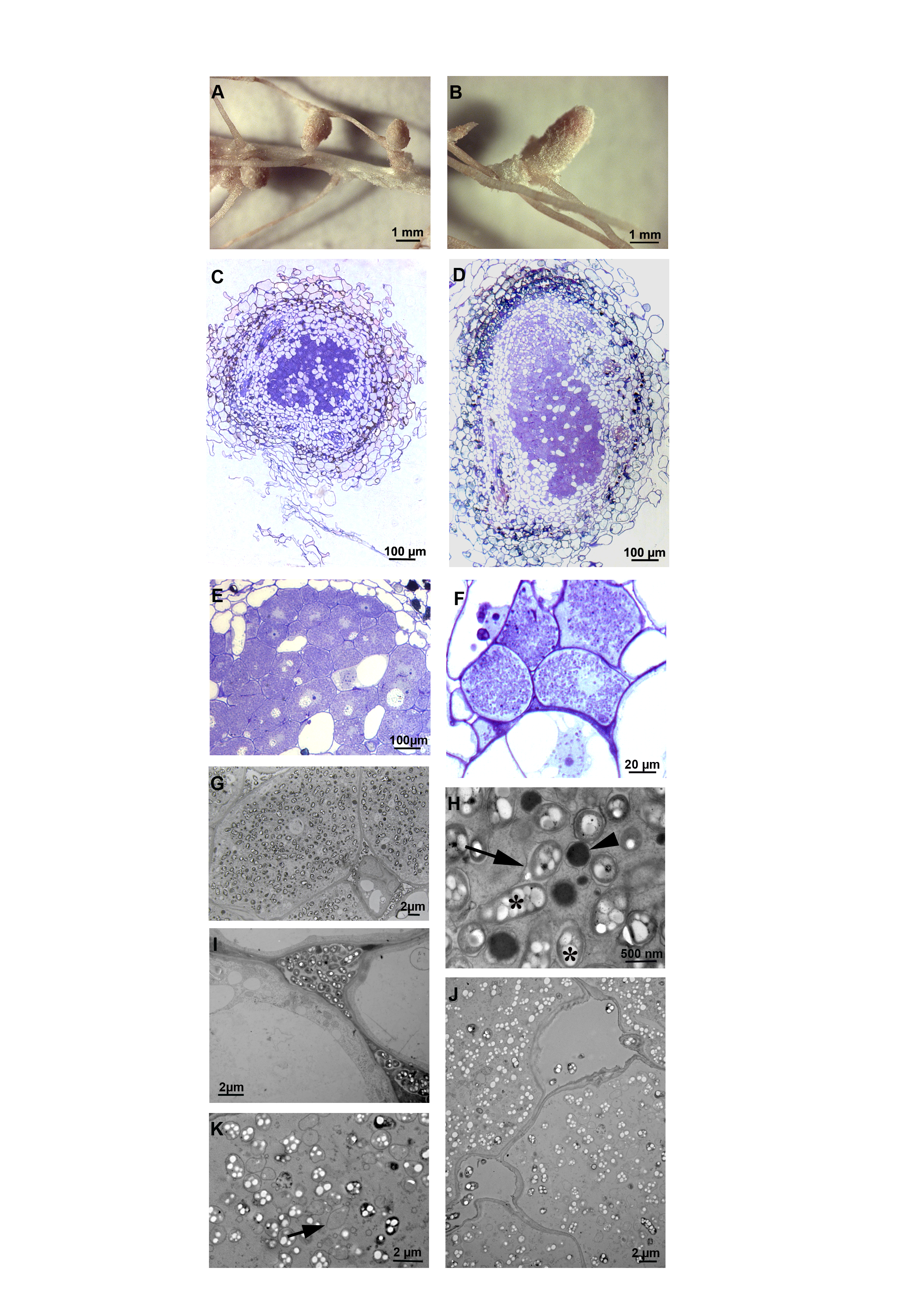

Supplement: Figure S8 — Nodulation and intracellular infection of M. pudica by hrpG chimeric mutant CBM664 (A and C) and evolved clones CBM212 (B, D, and F–K) and CBM349 (E). (A and B) Young nodules. (C and D) Nodule sections showing the infected zone. (E–G) Massive intracellular invasion in nodules. (G) Note the presence of bacteria in intercellular spaces. (H) Intracellular bacteria surrounded by a peribacteroid membrane forming typical symbiosomes (arrow). Osmophile material containing vesicles (arrowhead), probably involved in premature symbiosome degradation, were often associated with symbiosomes. PHB (Polyhydroxybutyrate) storage granules were present in bacteria (asterisks). (I) Infection pocket within intercellular space. (J and K) Premature senescence of 5-wk-old nodules with cytoplasmic structure desegregation of vegetal cells, loss of cell-to-cell contact, and numerous empty symbiosomes (arrow). (9.12 MB TIF) [file pbio.1000280.s008.tif]
