# Supplementary material for: Experimental Evolution of a Plant Pathogen into a Legume Symbiont
Source: PLoS Biol. 2010 Jan 12;8(1):e1000280. doi: 10.1371/journal.pbio.1000280 (PMC2796954; doi:10.1371/journal.pbio.1000280)
Supplement: Table S1 — Expression of a nodB::lacZ fusion in C. taiwanensis and chimeric Ralstonia in response to luteolin 15 µM. (0.03 MB DOC) [file pbio.1000280.s009.doc]

**Table S1**. **Expression of a *nodB::lacZ* fusion in *C. taiwanensis* and chimeric *Ralstonia* in response to luteolin 15 µM**

|  | β-galactosidase activity (Miller units) | |
| --- | --- | --- |
|  | no inducer | Luteolin (fold induction) |
| *C. taiwanensis* CBM132 | 79 +/- 14 | 3328 +/- 612 (x42) |
| Chimeric *Ralstonia* CBM134 | 31 +/- 7 | 1813 +/- 300 (x58) |

Values represent averages of three independent experiments
