# Supplementary material for: Experimental Evolution of a Plant Pathogen into a Legume Symbiont
Source: PLoS Biol. 2010 Jan 12;8(1):e1000280. doi: 10.1371/journal.pbio.1000280 (PMC2796954; doi:10.1371/journal.pbio.1000280)
Supplement: Table S2 — Number of mutations in evolved clones relative to the immediate ancestor CBM124GenR. (0.03 MB DOC) [file pbio.1000280.s010.doc]

**Table S2.** **Number of mutations in evolved clones relative to the immediate ancestor CBM124GenR**

| Clone | SNPs | Indels | Large deletions | Total (chrom1/chrom2/pRalta) |
| --- | --- | --- | --- | --- |
| CBM212 | 38 | 1 | 2 | 41 (17/19/5) |
| CBM349 | 27 | 1 | 2 | 30 (13/12/5) |
| CBM356 | 21 | 1 | 1 | 23 (5/13/5) |

Mutations were identified by analysis of the Illumina resequencing data (SNiPer Score>0.4). The number of mutations seems high as compared to other lab-evolution resequencing studies[1] for an estimated maximum 25 generations in the plant environment.

1. Velicer G, Raddatz G, Keller H, Deiss S, Lanz C, et al. (2006) Comprehensive mutation identification in an evolved bacterial cooperator and its cheating ancestor. Proc Natl Acad Sci U S A 103: 8107-8112.
