# Supplementary material for: Experimental Evolution of a Plant Pathogen into a Legume Symbiont
Source: PLoS Biol. 2010 Jan 12;8(1):e1000280. doi: 10.1371/journal.pbio.1000280 (PMC2796954; doi:10.1371/journal.pbio.1000280)
Supplement: Table S3 — List of primers. (0.06 MB DOC) [file pbio.1000280.s011.doc]

**Table S3**. List of primers

| **Gene/Region** | **Primer name** | **Primer sequence 5'-3'** | **Ampliconsize^a^** | **Use** |
| --- | --- | --- | --- | --- |
| AD1 | oCBM181 | NTCGASTWTSGWGTT |  | Degenerate primer for tailed PCR |
| AD4 | oCBM182 | AGWGNAGWANCAWAG |  | Degenerate primer for tailed PCR |
| Tn5 | oCBM183  oCBM184  oCBM185 | CAGAAGTTATCATGAACGTTACCA  TTCACTACCCTCTGATGAGATG  CGACCTGGTGGCGATAACTCA |  | Tn5 specific primers for tailed PCR |
| Tn5 insertion  point region | oCBM196  oCBM198 | ATCGTCCAGAAGCTGCAGGCA  GTTCTCCATGCGCAAGTCTTTC | 1985 bp | Amplification of the region surrounding the Tn5 insertion in strain CBM61 |
| 16S  (*C. taiwanensis*) | oCBM135  oCBM137 | CGGCAGCGCGGGCTTCGG  GGGATTTCACGCCTGTCTTATC | 545 bp | Verification CBM62 is not *C. taiwanensis* |
| 16S  (*R. solanacearum*) | oCBM138  oCBM140 | GGTGAAAGTGGGGGACCGC  GGATTTCACATCGGTCTTGCAC | 424 bp | Verification of CBM62 |
| *nifH* | oCBM170  oCBM24 | ATGGTAAAGGTGGCATCGGCA  GCATGCTGGACTACGTTGTCG | 636 bp | Verification of CBM62 |
| *nodB* promotor | oCBM203  oCBM211 | ccaagcttCCTCGCGACAGCTGCGCTC  aactgcagTGACTTTGGCATCACGTTCTCG | 401 bp | Cloning of the *nodB* promotor region in pCZ388 |
| *hrcS* | oCBM224  oCBM225 | TGCTCTACCTCGCCTTCATCGT  CAGCTGCTCCTGGTACTGGAG | 632 bp | Verification of *hrcS* inactivation in strain CBM142 |
| *hrcV* | oCBM214  oCBM215 | CTTTCCGTCGTTCCTGTTCGTC  TTGCGGATGGGCACCTGCTC | 740 bp | Verification of *hrcV* mutation in CBM125 |
| *hrcV* | oCBM763  oCBM764 | AGGAAACGCAGTGGATGCT  CCAAGCTCCTGGAACGAATA | 529 bp | Verification of the *hrcV* mutation in CBM356 |
| *hrpG* | oCBM622  oCBM623 | CGGAATTCACGTTTTCGTAATCGCCATC  GGGGTACCGTCTTCACGGTCTGCGAACT | 327 bp | Cloning of an *hrpG* upstream fragment in pCM184 |
| *hrpG* | oCBM624  oCBM625 | GCCGCGGTCTACAAGCTGCGCAAGAAA  GGTTAACAGCGTTGAAACCGTCAAATC | 401 bp | Cloning of an *hrpG* downstream fragment in pCM184 |
| *hrpG* | oCBM622  oCBM625 | CGGAATTCACGTTTTCGTAATCGCCATC  GGTTAACAGCGTTGAAACCGTCAAATC | 1000 bp | Verification of the *hrpG* mutations in CBM212 and CBM349 |
| *hrpG* | oCBM427  oCBM645 | AAGGCTTCGACGTCATCATC  CAAATCCAATGTGGAGATCG | 1500 bp | Verification of the *hrpG* deletion in CBM664 and Tn5 insertion in CBM663 |
| *popF1* | oCBM226  oCBM227 | ACCTGTTCTTCAGCTTCGGCGAT  GGGTGGTTGTCCGCGAACTTG | 693 pb | Verification of the *popF1* inactivation in CBM145 |
| *popF2* | oCBM228  oCBM229 | CGAACCCTCCCGACCTTCCGT  TTGATCTTGTCCTGGATCGACTG | 639 bp | Verification of the *popF2* inactivation in CBM145 |
| Rsp0125 | oCBM494  oCBM495 | ggaattcCAATTGTTCCAAGCGATGAA  catgccatggCGAAACGGAGGATAATTCCA | 547 bp | Cloning of a Rsp0125 fragment in pCM184 |
| Rsp0157 | oCBM496  oCBM497 | tccccgcggCGGGCTGATCAATCACATTT  cgagctcCTTCGTCCTCCAGCAGGTT | 514 bp | Cloning of a Rsp0157 fragment in pCM184 |
| Rsp0125-Rsp0157 | oCBM508  oCBM511 | CGCTATGGTGAGGGATCAGT  CTCGCCTTCCGAGTAATACG | 33000 bp | Verification of the 30 kb-deletion in strain CBM212, CBM349 CBM356 and CBM351 |
| Rsp1236-*lacZ* | oCBM505  oCBM326 | ggggtaccCTAAAAAGCGGCACCTTCTC  AAGGGGGATGTGCTGCAAGG | 800 bp | Verification of pCZ367 insertion in Rsp1236 in strain CBM206 |

^a^Expected size from wt strains GMI1000 or LMG19424
