# Supplementary material for: Experimental Evolution of a Plant Pathogen into a Legume Symbiont
Source: PLoS Biol. 2010 Jan 12;8(1):e1000280. doi: 10.1371/journal.pbio.1000280 (PMC2796954; doi:10.1371/journal.pbio.1000280)
Supplement: Table S4 — Characteristics of raw sequencing data output by the Illumina Genome Analyzera and SNiPer primary resultsb for the strains under study. (0.03 MB DOC) [file pbio.1000280.s012.doc]

**Table S4: Characteristics of raw sequencing data output by the Illumina Genome Analyzer ^(a)^ and SNiPer primary results ^(b)^** for the strains under study.

| **Strain** | **Number of reads^(a)^** | **Total coverage^(a)^** | **Number of mapped reads^(b)^** | **Effective coverage^(b)^** |
| --- | --- | --- | --- | --- |
| **CBM124GenR** | 10200738 | 57.7 | 7159703 | 40.5 |
| **CBM212** | 11340224 | 64.1 | 7800016 | 44.1 |
| **CBM349** | 10031276 | 56.7 | 5911623 | 33.4 |
| **CBM356** | 11191658 | 63.3 | 8315164 | 47.0 |
